# Comparative and Joint Analysis of Two Metagenomic Datasets from a Biogas Fermenter Obtained by 454-Pyrosequencing

**DOI:** 10.1371/journal.pone.0014519

**Published:** 2011-01-26

**Authors:** Sebastian Jaenicke, Christina Ander, Thomas Bekel, Regina Bisdorf, Marcus Dröge, Karl-Heinz Gartemann, Sebastian Jünemann, Olaf Kaiser, Lutz Krause, Felix Tille, Martha Zakrzewski, Alfred Pühler, Andreas Schlüter, Alexander Goesmann

**Affiliations:** 1 Computational Genomics, Center for Biotechnology (CeBiTec), Bielefeld University, Bielefeld, Germany; 2 Roche Diagnostics GmbH, Penzberg, Germany; 3 Department of Genetechnology/Microbiology, Bielefeld University, Bielefeld, Germany; 4 Department of Periodontology, University Hospital Münster, Münster, Germany; 5 Division of Genetics and Population Health, Queensland Institute of Medical Research, Herston, Australia; 6 Institute for Genome Research and Systems Biology, Center for Biotechnology (CeBiTec), Bielefeld University, Bielefeld, Germany; Cairo University, Egypt

## Abstract

Biogas production from renewable resources is attracting increased attention as an alternative energy source due to the limited availability of traditional fossil fuels. Many countries are promoting the use of alternative energy sources for sustainable energy production. In this study, a metagenome from a production-scale biogas fermenter was analysed employing Roche's GS FLX Titanium technology and compared to a previous dataset obtained from the same community DNA sample that was sequenced on the GS FLX platform. Taxonomic profiling based on 16S rRNA-specific sequences and an Environmental Gene Tag (EGT) analysis employing CARMA demonstrated that both approaches benefit from the longer read lengths obtained on the Titanium platform. Results confirmed *Clostridia* as the most prevalent taxonomic class, whereas species of the order *Methanomicrobiales* are dominant among methanogenic *Archaea*. However, the analyses also identified additional taxa that were missed by the previous study, including members of the genera *Streptococcus*, *Acetivibrio*, *Garciella*, *Tissierella*, and *Gelria*, which might also play a role in the fermentation process leading to the formation of methane. Taking advantage of the CARMA feature to correlate taxonomic information of sequences with their assigned functions, it appeared that *Firmicutes*, followed by *Bacteroidetes* and *Proteobacteria*, dominate within the functional context of polysaccharide degradation whereas *Methanomicrobiales* represent the most abundant taxonomic group responsible for methane production. *Clostridia* is the most important class involved in the reductive CoA pathway (Wood-Ljungdahl pathway) that is characteristic for acetogenesis. Based on binning of 16S rRNA-specific sequences allocated to the dominant genus *Methanoculleus*, it could be shown that this genus is represented by several different species. Phylogenetic analysis of these sequences placed them in close proximity to the hydrogenotrophic methanogen *Methanoculleus bourgensis*. While rarefaction analyses still indicate incomplete coverage, examination of the GS FLX Titanium dataset resulted in the identification of additional genera and functional elements, providing a far more complete coverage of the community involved in anaerobic fermentative pathways leading to methane formation.

## Introduction

The fraction of renewable energy forms for energy supply is constantly increasing since fossil fuels are running short and energy production from fossil fuels brings about emissions of the greenhouse gas carbon dioxide which has implications on the climate. In this context the production of biogas by means of fermentation of biomass becomes more and more important because biogas is regarded as a clean, renewable and environmentally compatible energy source [1], [2]. Moreover, generation of energy from biogas relies on a balanced carbon dioxide cycle. In Germany biogas is mainly produced from energy crops such as maize and liquid manure in medium-sized agricultural biogas plants [1]. The microbiology of biogas formation from organic matter is complex and involves interaction of different microorganisms. In the first step of the digestion process, organic polymers of the substrate such as cellulose, other carbohydrates, proteins and lipids are hydrolysed to low-molecular weight compounds [3]–[5]. Cellulolytic *Clostridia* and *Bacilli* are among other bacteria important for this step. Subsequently, fermentative bacteria convert low-molecular weight metabolites into volatile fatty acids, alcohols, and other compounds which are then predominantly metabolised to acetate, carbon dioxide and hydrogen by syntrophic bacteria [6]–[11]. These latter compounds are in fact the substrates for methane synthesis which is accomplished by methanogenic *Archaea* [12], [13]. Hydrogenotrophic *Archaea* are able to reduce carbon dioxide to methane using hydrogen as an electron donor, whereas aceticlastic *Archaea* convert acetate to methane [14]–[18]. The biochemistry and enzymology of methanogenesis is well known for model organisms, but the functioning of biogas-producing microbial communities on the whole is insufficiently explored. Community structures of biogas-producing microbial consortia were analysed for different systems and settings including a thermophilic municipal biogas plant [19], a thermophilic anaerobic municipal solid-waste digester [20], thermophilic upflow anaerobic filter reactors [21], a completely stirred tank reactor fed with fodder beet silage [22], a two-phase biogas reactor system operated with plant biomass [23], an anaerobic sludge digester [24], mesophilic anaerobic chemostats [25], [26], a packed-bed reactor degrading organic solid waste [27] and many other habitats. Most of these studies were based on the construction of 16S rRNA clone libraries and subsequent sequencing of individual 16S rRNA clones. The resulting nucleotide sequences were then taxonomically and phylogenetically classified to deduce the structure of the underlying community. Also, *mcrA* clone libraries were used to elucidate methanogenic archaeal communities of different habitats [28]–[32]. The *mcrA* gene encodes the alpha subunit of methyl-coenzyme M reductase representing the final enzyme in the methanogenesis pathway. Since *mcrA* is present in all methanogenic *Archaea* analysed so far, it serves as a phylogenetic marker for this group of *Archaea*. Usually, analyses of *mcrA* and 16S rRNA clone libraries do not cover the whole complexity of the respective habitats since sequencing can only be done for limited numbers of clones. Moreover, results of clone library analyses are always biased by the choice of primers that are used for amplification of marker gene fragments and cloning efficiencies. In recent years, microbial communities have been studied on the basis of their metagenomes which became accessible by applying high-throughput sequencing technologies. Recently, the first metagenome sequencing approach for a biogas-producing community was described [33]. Community DNA isolated from a production-scale biogas plant fed with maize silage, green rye and low amounts of chicken manure was sequenced on the Genome Sequencer FLX platform which resulted in 142 million base pairs of sequence information. Bioinformatic methods were employed to deduce the taxonomic composition and functional characteristics of the intrinsic biogas community [34]. Analysis of the community revealed *Clostridia* as the most prevalent phylogenetic class, whereas species of the order *Methanomicrobiales* are dominant among methanogenic *Archaea*.

Similar results were obtained by parallel construction of 16S rRNA and *mcrA* amplicon libraries and subsequent sequencing of cloned fragments [35]. Moreover, bioinformatics results indicated that *Methanoculleus* species play a dominant role in methanogenesis and that *Clostridia* are important for hydrolysis of plant biomass in the analysed fermentation sample. Rarefaction analysis of the metagenome data showed that the sequencing approach was not carried out to saturation. Sufficient coverage of non-abundant microbial groups in the fermentation sample would require deeper sequencing. Therefore, the available total community DNA preparation from the biogas fermentation sample was additionally sequenced on the GS FLX Titanium platform, which provides longer read lengths and increased throughput compared to the GS FLX platform. This paper describes an integrated analysis of the GS FLX and the GS FLX Titanium datasets with the objective to deepen the knowledge on the taxonomic structure and composition of a microbial community involved in biogas production within an agricultural, production-scale biogas plant. Moreover, the described analysis intends to elucidate the metabolic capacity of the community, functional roles of specific microorganisms and key organisms for the biogas production process.

## Methods

### Total community DNA preparation and sequencing

Total community DNA of a biogas fermentation sample obtained from an agricultural biogas plant was prepared by a CTAB-based DNA-isolation method [36] as described recently. More detailed information on the origin of the fermentation sample is given in a previous publication [33]. An aliquot of the DNA preparation that was recently used for whole genome shotgun sequencing on the Genome Sequencer (GS) FLX platform now served as template DNA for sequencing on the GS FLX Titanium platform. The sequencing library was constructed according to the protocol of the GS Titanium General Library Prep Kit (Roche Applied Science). After titration of the library using the GS Titanium SV emPCR Kit, a full sequencing run was carried out on the GS FLX Titanium platform.

### Data normalization

To assess the overall comparability of the pyrosequencing datasets obtained from the GS FLX and Titanium platforms, the average GC content of all reads was determined for each dataset. For this, various Perl scripts were developed to determine the overall and individual GC content of the obtained reads. Results were visualized using the statistical computing software R [37].

To normalize the data with respect to the observed GC bias (see Results), an outlier detection approach was applied and the endpoints of the linear phases were determined; sequences longer than the computed thresholds were excluded. A linear regression was calculated for the GC plot beginning with 30 data points starting at the 100 bp position to exclude the portion of the dataset with high variance in GC content. In the next step, externally studentized residuals [38] were computed for the linear regression. Each data point was inspected whether it deviated from the linear trend using a Student-t-test. A data point was regarded as being an outlier if the 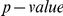 of the studentized residuals test was below 0.05. If ten outliers in a row were found, the first one of these is representing the end of the linear phase and was taken as threshold value for filtering of the dataset.

To rule out sequencing errors as the reason for the observed decreasing GC content of the longer reads, the analysis was repeated for publicly available pyrosequencing datasets from both metagenome as well as genome sequencing projects which were obtained from NCBI's Short-Read Archive (SRA). The effect in question could be observed for all metagenome datasets, where the DNA fragments from a mixture of organisms have a broad distribution concerning their GC contents. Single genome sequencing projects, on the other hand, did not show this effect, thereby ruling out sequencing errors as an alternative explanation (see File S1). The comparably narrow GC distribution in the sequence data from single genome projects does not reveal this effect, and while backfolding might limit maximum obtainable read length, similar filtering steps would not be a prerequisite before e.g. subsequent assembly.

A recent study [39] described the occurence of artificially created duplicate reads in datasets generated using the pyrosequencing method. It is assumed that the duplication of individual DNA fragments occurs during the emulsion PCR reaction, which is a step of the library preparation. These nearly identical sequences might lead to inflated estimates of functional genetic elements or introduce an artificial shift in taxonomic profiles, unless they are filtered from the dataset. In this study, these duplicates were removed using the cdhit-454 [40] program, which filters almost identical reads beginning at the same position. Each dataset was processed separately using the accurate mode (option ‘-g 1’) of the software.

### Identification and taxonomic classification of 16S rRNA fragments

A BLAST [41] search versus the RDP database (Release 10.10) was conducted to identify reads carrying fragments of 16S rRNA genes. Since BLAST excludes regions with low sequence complexity by default, the sequence complexity filter was explicitly disabled (option ‘-F F’). Alignments with an E-value of 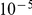 or better and a minimum length of 50 bp were extracted and processed using the RDP classifier [42], which employs a naive bayesian classifier to assign the sequences to taxonomic categories. Only 16S rRNA fragments with at least 80% assignment confidence were considered.

### Taxonomic classification of Environmental Gene Tags (EGTs)

Metagenomic sequences were filtered using a BlastX search against the Pfam database [43]. Reads without a hit were additionally scanned for conserved protein domains by conducting a search for protein family members using Pfam HMMs. Community sequence reads that were predicted to contain fragments of genes (environmental gene tags, EGTs) were subsequently classified on different taxonomic ranks based on a phylogenetic tree of the metagenomic read itself and the matching Pfam protein family member sequences. A full description of the CARMA pipeline can be found in the original publication [44].

Here, an unpublished improved version of CARMA was applied to process both pyrosequencing datasets using the default settings. For reads containing more than one EGT and therefore more than one classification, the taxonomic classifications were merged into a single one that was determined as the lowest common ancestor of all classifications. While this approach decreases the number of classifications on the lower taxonomic ranks, it simultaneously eliminates contradicting classifications and reduces the influence of potential mis-classifications.

### Community participation in substrate decomposition, fermentation and methane production

For further analysis both datasets were merged together, as no significant differences in the taxonomy as well as in the related enzyme composition could be observed. Orthologous groups of genes were retrieved by comparing the sequences to the eggNOG database [45]. For this, sequences were assigned to COGs and NOGs using BlastX with an E-value cutoff of 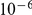 and annotated according to their best hit. Additionally, taxonomic information was added using the results obtained from the CARMA pipeline. Enzymes relevant for biochemical processes were identified with the help of MetaCyc [46] and the corresponding COGs (Clusters of Orthologous Groups) were selected and grouped. The conclusive tree construction based on the NCBI taxonomy [47], visualisation and data analysis were performed using unpublished software.

### Identification of *Methanoculleus* variants

To confirm the presence of different *Methanoculleus* species in the biogas fermenter, 16S rRNA fragments that were identified by a homology search and classified as belonging to the genus *Methanoculleus*, as described above, were assembled into longer contigs.

Here, the 16S rRNA nucleotide sequence from *Methanoculleus bourgensis* (GenBank accession AY196674) was used as an assembly template. The pyrosequencing reads were aligned to the reference sequence using ‘align0’ from the FASTA3 package [48] and then clustered into several groups based on their SNP (single nucleotide polymorphism) content. Afterwards, each of the resulting groups of reads was separately assembled employing Roche's GS de novo Assembler software. To put the resulting consensus sequences into context, a phylogenetic characterization was performed. Together with other 16S rRNA sequences obtained from GenBank, a multiple alignment of all sequences was computed using the MUSCLE tool [49] and a phylogenetic tree was generated with the fdnapars program [50], which implements the DNA parsimony algorithm.

### Mapping of pyrosequencing reads to *Methanoculleus marisnigri* JR1

To identify the overall sequence similarity between the metagenomic reads and the genome of *Methanoculleus marisnigri* JR1, all reads were mapped to the published DNA sequence of this genome [51]. In a previous study [34], reads were aligned to reference sequences using BLAST with rather relaxed settings (E-Value <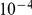, aligned region >100 bp, sequence identity >80%). Since this approach did not account for the relative fraction of a read taking part in an alignment, in this study the gsMapper software from Roche was employed. Coverage information for the genome and individual genes was extracted from the gsMapper output using various Perl scripts; the results for gene coverage were normalized and divided into two groups based on the prediction found in the HGT-DB [52], a database of procaryotic genomes that uses different statistical properties of coding sequences to predict whether they may have been acquired by horizontal gene transfer. While GC content is one of these parameters, several others such as codon and amino acid usage are also used. The R statistical software was employed to compute the kernel density estimates for both groups; for this, an Epanechnikov kernel with a bandwith of 0.4 was used.

### Detection of hydrogenase gene fragments

To analyse the occurrence of metagenomic reads encoding hydrogenases or proteins involved in hydrogen uptake systems, corresponding genes annotated as hydrogenases or hydrogen uptake systems were extracted from the reference genome of *Methanoculleus marisnigri* JR1 [51]. Related gene fragments in the metagenome datasets were identified by a BLAST search using an e-value cutoff of 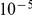 and a disabled sequence complexity filter (option ‘-F F’); for each sequence, only the best BLAST hit was considered.

### Biodiversity and Rarefaction analysis

To gain an overview of the biodiversity of the studied microbial community, the Shannon index was computed based on 16S rRNA fragments classified on rank genus with at least 80% confidence as
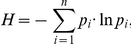
where 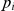 is the relative abundance of sequences assigned to genus 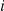, and 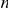 denotes the total number of different genera.

Additionally, a rarefaction analysis was employed to assess the coverage of the microbial community by the datasets. The number of genera that would be observed for different sample sizes was estimated using Analytic Rarefaction (version 1.3). Rarefaction curves were obtained by plotting the sample sizes versus the estimated number of genera.

A separate rarefaction analysis was conducted to assess the coverage of the collective gene content of the microbial community. The number of Pfam protein families that would be identified in metagenomes of different sizes was estimated using Analytic Rarefaction. Rarefaction curves were generated by plotting the number of Pfam families versus the sample sizes.

### Data availability

Sequence data from both the GS FLX and Titanium run has been deposited at the NCBI Short Read Archive (SRA) under the accessions SRR030746.1 for the GS FLX and SRR034130.1 for the Titanium dataset. Assembled 16S rRNA consensus sequences of the different *Methanoculleus* variants have been submitted to the GenBank database (accessions GU731070 to GU731076).

## Results

### Sequencing of the biogas microbial community on the GS FLX Titanium platform and data preprocessing

**Figure 1:**
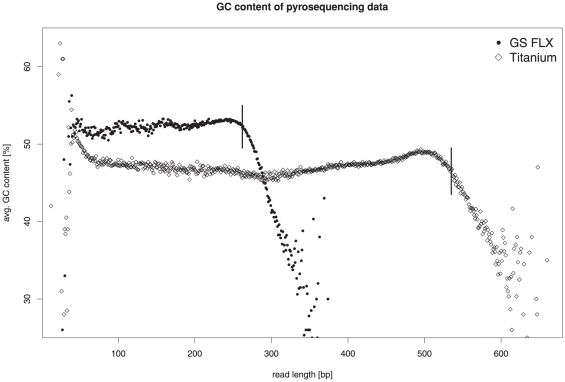
Read length and average GC content of pyrosequencing reads. A sharp decline in GC content can be seen once the read length exceeds a certain value. The vertical bars indicate the computed filtering thresholds for the GS FLX and the Titanium dataset, respectively.

Metagenomic DNA from a biogas-producing microbial community residing in the fermenter of an agricultural biogas plant fed with maize silage, green rye and low amounts of chicken manure was recently sequenced on the Genome Sequencer (GS) FLX platform [33]. This approach resulted in 616,072 sequence reads with an average read length of 230 bases, accounting for approximately 142 million bases sequence information. Analysis of the obtained sequence data revealed that sequencing was not carried out to saturation [34]. To achieve a deeper coverage of the intrinsic biogas community and to elucidate its whole complexity, the same community DNA preparation that was used for sequencing on the Genome Sequencer FLX was now sequenced on the GS FLX Titanium platform. The single Titanium run yielded 1,347,644 reads with an average length of 368 bases resulting in 495.5 million bases sequence information, which represents a 3.5-fold increase in coverage of the sample compared to the previous sequence dataset. Statistical data of the Titanium run are summarized and compared to the sequencing approach on the GS FLX platform in Table 1. Although aliquots of the same DNA sample were used for sequencing on both platforms, the average GC content of the reads generated on the GS FLX platform was determined as 51.7%, while the GC content of the Titanium reads was determined as only 47.4%. Also, the average GC content for different read lengths was analysed. Results showed that GC content and obtained read length clearly correlate for both GS FLX and the Titanium platform. The applied sequencing methods did not only generate reads with different average levels of GC content, but also both pyrosequencing platforms show a significant decline in GC content once the read length exceeds a certain value (Figure 1).

**Table 1:** Sequencing results.

	number of reads	number of bases	avg. read length
GS FLX	616,072	141,685,079	230.0 bp
Titanium	1,347,644	495,506,659	367.7 bp
ratio Titanium/GS FLX	2.19	3.50	1.60

Overview of sequence data obtained from the studied biogas fermenter employing different pyrosequencing technologies.

While the differences in average GC content can be explained by variations in the sequencing chemistry and protocol used for sequencing on the GS FLX and Titanium platforms, the sharp decline of the GC content present in the longer reads is most probably caused by backfolding. It is assumed that the GC-rich parts of the synthesized ssDNA are forming stable secondary structures during the emulsion PCR process, which subsequently leads to a termination of the PCR reaction and only the non-folded part of the DNA fragment being amplified. On the other hand, DNA fragments with lower GC content are not affected by this problem and can be amplified over their full length.

Previous studies have already identified GC biases in reads generated on the Illumina platform [53], but no such findings have yet been reported for Roche's pyrosequencing method. Such biases caused by the sequencing chemistry and protocol are likely to remain unnoticed in many cases, where research is focused on analysis of single datasets with no additional data available for comparison. In the context of metagenome studies, these effects can easily lead to a distortion of results, where e.g. taxonomic profiles would underestimate the relative abundance of GC-rich organisms.

In this study, both datasets were filtered to minimize the impact of the identified GC bias and exclude the artificial duplicate sequences. The endpoints of the linear phases of the GC plot were determined at 262 bp for the GS FLX and 535 bp for the Titanium dataset; subsequent removal of sequences longer than the computed thresholds resulted in the exclusion of 169,569 (27.52%) of the GS FLX reads and 26,795 (1.98%) sequences from the Titanium dataset, which is only marginally affected. This shows a significant improvement regarding GC bias for the GS FLX Titanium chemistry compared to the previous GS FLX chemistry.

After the removal of technical replicates, 407,558 reads from the GS FLX dataset and 1,019,333 of the Titanium reads passed all filtering steps and are suitable for further analysis. Throughout this study, these normalized datasets were used for the computation of relative abundances of either taxonomic groups or the functional characterization.

### Taxonomic composition of the microbial community based on 16S rRNA analysis

The DNA sequence of the 16S rRNA gene has found wide application for taxonomic and phylogenetic studies. It is highly conserved between both *Archaea* and *Bacteria*, but also contains hypervariable regions that can be exploited for accurate taxonomic assignments. Using the previously filtered pyrosequencing reads to avoid a distortion of taxonomic profiles, a BLAST homology search using the RDP database was performed. In this step, 616 16S rRNA fragments with an average length of 159.5 bp were identified in the GS FLX dataset, while 2,709 fragments with an average length of 245.2 bp from the Titanium reads could be detected. The differing number of 16S rRNA sequences identified in the GS FLX dataset in comparison to the findings of a previous study [34] can be explained by the data normalization step as described above. Furthermore, the ARB database [54] was used instead of the RDP database. All identified 16S rRNA sequences were taxonomically classified by means of the RDP classifier [42]. For all taxonomic ranks except domain, the RDP classifier was able to assign a larger fraction of 16S rRNA fragments from the Titanium dataset with at least 80% confidence than from the FLX data. While 18.2% of the 16S rRNA fragments from the GS FLX reads could be classified on rank order, 25.3% of the Titanium 16S rRNA fragments were assigned on this rank, showing that the RDP classifier benefits from the longer reads generated on the Titanium platform. On rank genus, 6.0% of the GS FLX 16S rRNA fragments and 10.6% of the Titanium 16S rRNA fragments could be assigned. Nevertheless, it has to be noted that only a low fraction of pyrosequencing reads actually contains 16S rRNA fragments (0.15% of the filtered GS FLX reads; 0.26% of the filtered Titanium reads). While 16S rRNA genes are reliable phylogenetic markers, the low number that could be detected in the present metagenome datasets gives only a broad overview of the most abundant taxonomic groups and may be insufficient to obtain a detailed picture of the taxonomic composition of a metagenome.

### Taxonomic classification of Environmental Gene Tags (EGTs)

**Figure 2:**
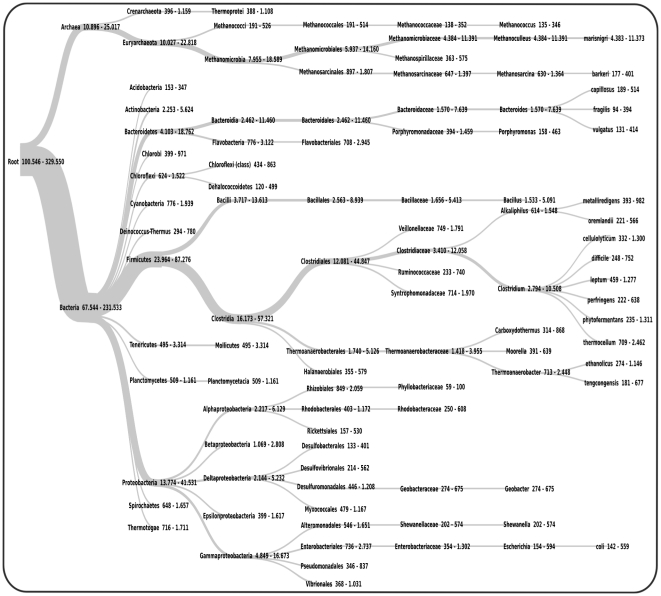
Characterization of the GS FLX and Titanium datasets based on the taxonomic classification of Environmental Gene Tags (EGTs). Displayed are only the most abundant taxa among *Bacteria* and *Archaea* lineages at various taxonomic ranks. For each group, the first number represents the number of assigned EGTs from the GS FLX dataset, the second number the EGTs from the Titanium dataset, respectively. Due to the different number of reads obtained from each sequencing platform, the amount of EGTs listed for the Titanium dataset is typically the fourfold of the number listed for the GS FLX dataset.

**Figure 3:**
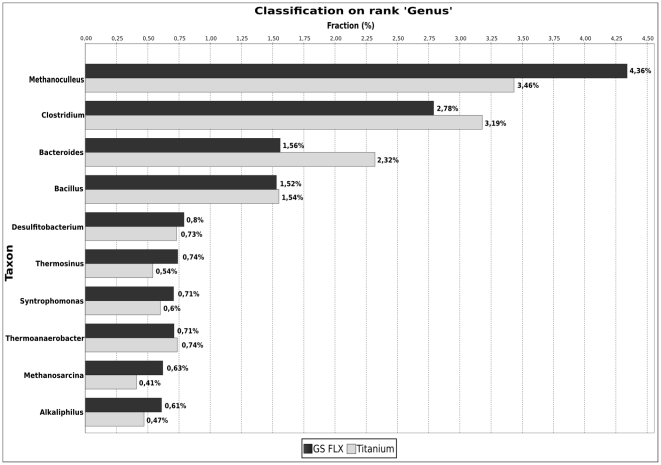
Comparison of taxonomic profiles on rank genus. The taxonomic profiles for the GS FLX (black bars) and the Titanium (lightgray bars) datasets were computed employing the CARMA pipeline. The percentage values correspond to the total amount of reads in the filtered datasets; included are the ten most abundant genera.

One of the major limitations of the taxonomic classification of 16S rRNA fragments is the low fraction of reads actually containing 16S rRNA specific sequences. The CARMA software [44] overcomes this limitation as it is based on the identification of environmental gene tags (EGTs) in community sequences using profile hidden Markov models (pHMMs) and the phylogenetic deduction of the taxonomic origin of these fragments. The major strength of this approach is the high accuracy of pHMMs for the detection of short functional segments of Pfam protein family members. The search for conserved Pfam protein fragments resulted in a total of 100,546 identified EGTs (25% of all reads) for the GS FLX dataset and 329,550 (32% of all reads) for the Titanium dataset, respectively. At the taxonomic rank superkingdom, 88,528 of the GS FLX reads (22%) and 290,008 of the Titanium reads (28%) could be successfully classified. The fact that a higher fraction of Titanium reads could be assigned indicates that CARMA as well benefits from the increased read lengths generated by the Titanium platform. The community composition as deduced from EGTs identified in both datasets is almost identical: The majority of EGTs was classified as belonging to the superkingdoms *Bacteria* (GS FLX: 67%; Titanium: 70%) and *Archaea* (11% and 8%, respectively). This conformity of taxonomic composition was also observed for almost all the prevalent and rare taxa at other taxonomic ranks (Figure 2). Despite this general accordance, there are some noteworthy differences between the GS FLX and Titanium datasets. While the summarized percentage of identified taxa at rank genus is roughly the same in both datasets, the actual abundance of specific genera differs between both datasets (Figure 3). A higher amount of variation exists especially for the most abundant genera between the two datasets: Both datasets support *Methanoculleus* as the most abundant genus, but a higher fraction of GS FLX reads was assigned to this genus than from the Titanium reads (4.36% of the GS FLX dataset; 3.46% of the Titanium data). For the genus *Clostridium*, this ratio is reversed: only 2.78% of the GS FLX reads, but 3.19% of the Titanium sequences were allocated to this genus. Similar differences can also be noted for *Bacteroides* and *Bacillus*. Even though differences exist between the two taxonomic profiles, results based on analysis of the Titanium dataset can be considered more reliable since they were deduced from a larger amount of sequence information. The discrepancy of the taxonomic profile for the GS FLX dataset as shown here in contrast to previously reported results [34] is due to the applied GC filtering step and adjustments within the CARMA software. Applying an unpublished, enhanced version of CARMA to the unfiltered GS FLX dataset, EGTs were identified for 167,134 (32%) of the reads, while only 133,337 (22%) EGTs were reported in the previous study.

### New genera identified in the GS FLX Titanium dataset

Due to the deeper coverage of the metagenome by the dataset obtained on the GS FLX Titanium system, new genera were identified in the corresponding taxonomic profile (see Table 4.1, File S1). These include *Streptococcus*, *Acetivibrio*, *Garciella*, *Tissierella*, *Gracilibacter*, *Gelria*, *Dysgonomonas* and *Arcobacter* to mention just a few. *Streptococcus* species were previously detected in different anaerobic habitats, especially in a mesophilic hydrogen-producing sludge and a glucose-fed methanogenic bioreactor [55]–[57]. In the latter habitat, an acetate- and propionate-based food chain was prevalent but the specific functions of the *Streptococcus* members dominating the bioreactor are not known [55]. Sequences related to the genus *Acetivibrio* (*Firmicutes*) were recently recovered from a community involved in methanogenesis utilizing cellulose under mesophilic conditions [58]. *Acetivibrio* species most probably play a role in cellulose degradation [58], [59]. Species of the genera *Garciella*, *Tissierella*, *Gracilibacter* and *Gelria* (all *Firmicutes*) are also adapted to anaerobic habitats where they are involved in different fermentative pathways [60]–[65]. Interestingly, a reference species of the genus *Geleria*, namely *G. glutamica*, was isolated from a propionate-oxidizing methanogenic enrichment culture and represents an obligately synthrophic, glutamate-degrading bacterium that is able to grow in co-culture with a hydrogenotrophic methanogen [65]. In this context it should be mentioned that hydrogenotrophic methanogens are dominant in the fermentation sample analysed in this study. The genus *Dysgonomonas* (see Table 4.1, File S1) belongs to the family *Porphyromonadaceae* of the order *Bacteroidales*. Members of the genus *Dysgonomonas* were *inter alia* isolated from stool samples and are able to ferment glucose resulting in the production of acids [66]. Likewise, bacteria of the genus *Alkaliflexus* also cluster within the order *Bacteroidales* and represent anaerobic saccharolytic organisms [67]. Other genera such as *Arcobacter* were indeed found in an anaerobic community digesting a model substrate for maize but seem to play no dominant role in degradation of polysaccharides [68]. Some of the genera listed in Table 4.1 (File S1) have not been described for anaerobic, methanogenic consortia so far and hence their involvement in the biogas production process remains unknown. In summary, the more detailed taxonomic analysis presented in this study also revealed less-abundant genera that were missed in the previous taxonomic profile for the same community. Some of the newly identified genera presumably are of importance for the biogas production process.

### Community participation in substrate decomposition, fermentation and methane production

As described in a previous study, *Firmicutes* and *Methanomicrobiales* play a crucial role in hydrolysis, acetogenesis and methanogenesis representing key steps in anaerobic degradation of plant biomass [34]. In this study corresponding pathways are investigated in more detail using the combined dataset consisting of the GS FLX and newly acquired Titanium reads, and an elaborated methodology to identify key organisms involved in the above mentioned processes. For this purpose all reads were classified according to Cluster of Orthologous Groups (COG) categories to infer the functional potential of the underlying community. In a second step, obtained COG results were annotated with the taxonomic information generated by the CARMA software. This approach led to the identification of 292,782 reads (about 21% of the dataset) for which both functional as well as taxonomic information could be retrieved. Moreover, a subset of COG entries representing (a) the process of ‘polysaccharide degradation’, (b) ‘acetogenesis’ and (c) the ‘methanogenesis’ step within the fermentation process were chosen for a more detailed taxonomic analysis (see File S1). Even though not all reads classified into the selected COG categories may actually represent the pathways in the focus of this approach, this analysis provides insights into the relevance of different taxonomic groups for the hydrolysis, acetogenesis, and methanogenesis steps in fermentation of biomass. Misallocation of reads to these processes can be due to the fact that some COG entries include enzymes involved in different, but functionally related pathways.

**Figure 4:**
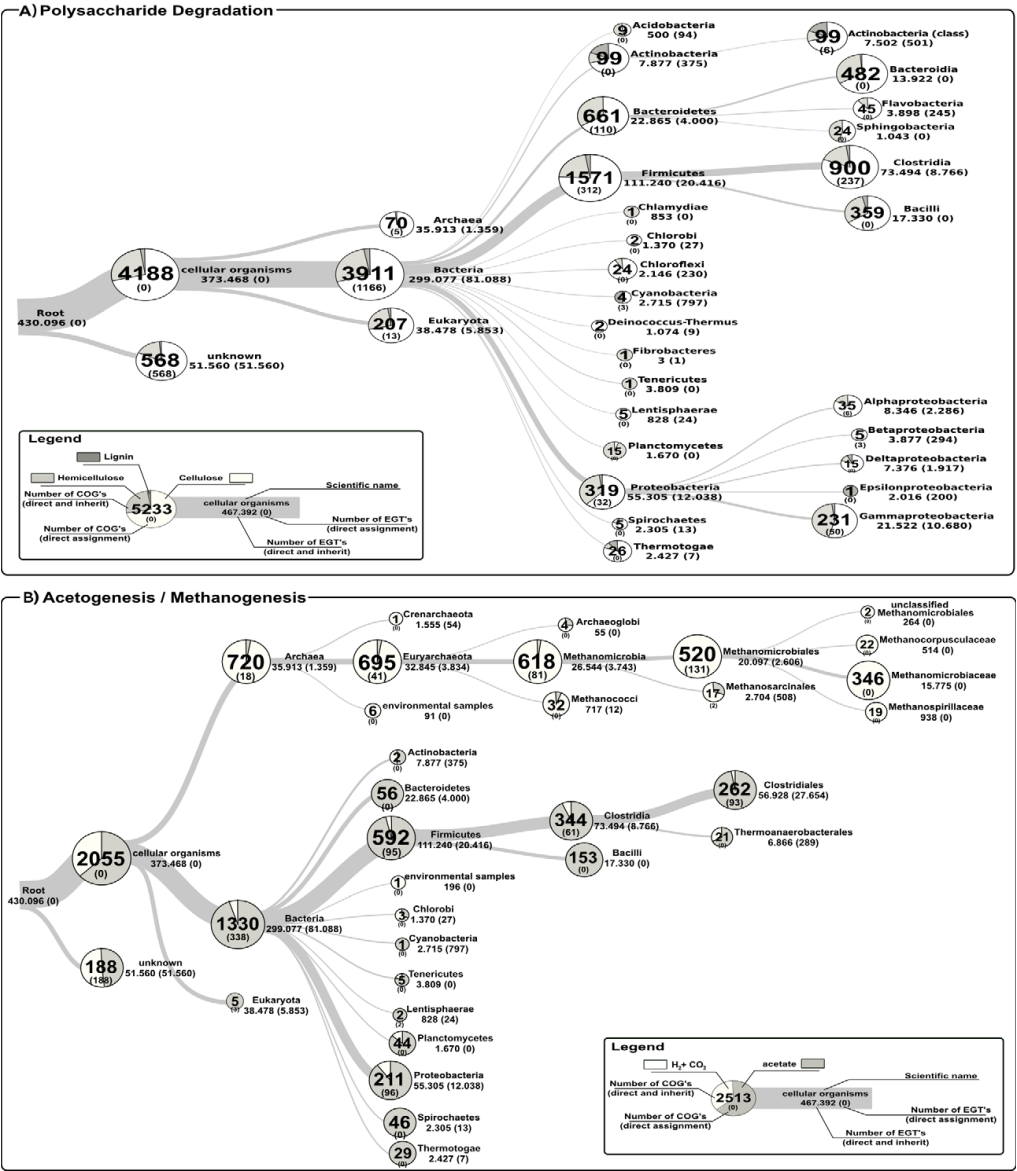
Taxonomic and physiological overview of relevant members in ‘polysaccharide degradation’ (a) and ‘acetogenesis/methanogenesis’ (b). The trees are based on reads classified into the NCBI taxonomy by the CARMA software. For each taxonomic group the underlying number of reads is given. Numbers in brackets refer to the amount of reads which could not be classified at corresponding lower taxonomic ranks. Associated COG entries are depicted as pie charts, where the interpretation of the numbers is equivalent.

One of the first steps in the fermentation process is the breakdown of polysaccharide components of plant cell material. Especially, the cell wall polymers cellulose, hemicellulose and pectin constitute a high amount of the carbon and energy resource that is available for bacteria in the fermentation sample. Accordingly, hydrolysis of plant biomass is considered to be the rate-limiting step in biogas production. Based on the assignment to COG entries (see File S1), a total of 4,762 reads were classified as potentially coding for enzymes involved in the degradation of complex polymers, namely cellulose, hemicellulose, and lignin (Figure 4a). Reads allocated to the cellulose degradation process account for 71% of the 4,762 reads. Hence, they constitute the most prevalent group followed by reads predicted to encode hemicellulose degrading enzymes (27%). As expected, reads related to lignin degradation (2%) are only rarely found. This is due to the fact that lignin degradation predominantly occurs under aerobic conditions, whereas fermentation of plant biomass for biogas production is an anaerobic process. Most of the reads (1,571) assigned to the ‘polysaccharide degradation’ context originate from members of the *Firmicutes* making this phylum the most important one. Additionally, *Bacteroidetes* (661) and *Proteobacteria* (319) are involved in this process, since significant numbers of reads were grouped into these taxa. The phylum *Actinobacteria* includes a high fraction of reads encoding fragments of lignin degrading enzymes. This is in accordance with literature, since *Actinobacteria* are known to express different ligninases [69].

The reductive acetyl-CoA pathway (also called Wood-Ljungdahl pathway) is important for acetogenic bacteria to autotrophically produce acetate from hydrogen and carbon dioxide or carbon monoxide, respectively [70], whereas aceticlastic methanogens reversely use this pathway to generate methane from acetate. Several COG classifications representing the Wood-Ljungdahl and the hydrogenotrophic methanogenesis pathway were taxonomically analysed to distinguish between aceticlastic [16] and hydrogenotrophic methanogens [71]. The fact that the Wood-Ljungdahl pathway is used by acetogenic bacteria like *Clostridia* as well as methanogenic *Archaea* is shown in Figure 4b. To address the total number of COG hits representing methanogenesis, only *Archaea* with 720 assignments were taken into account for subsequent analysis. The order *Methanomicrobiales* (520) constitutes 72% of all COG hits relevant for methanogenesis assigned to the superkingdom *Archaea*. Thus, the order *Methanomicrobiales* is by far the most abundant taxonomic group producing methane using CO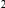 as a carbon source. Besides, it is noticeable that 25% of the reads assigned to *Methanomicrobiales* (131) could not be classified at lower taxonomic levels, indicating that either corresponding proteins originate from new taxa which are not represented in COG or highly conserved proteins shared by more than one taxon. Acetate as source for methane production seems to play a minor role, which is taxonomically indicated by the low fraction of *Methanosarcinales* (2,704 reads) within the group of known methanogens (27,693 reads). This observation correlates with the low fraction of hits (3.5%) indicative for acetate conversion to methane within the group of *Archaea* as well as the fact that reductive acetyl-CoA pathway enzymes are mainly found in the group of *Bacteria* (95%) with *Clostridia* as their most abundant class.

### Identification of several different *Methanoculleus* species

Analysis of the community participation in methanogenesis revealed that members of the order *Methanomicrobiales* are dominant among the methanogenic *Archaea*. Recently, the complete genome sequence of *Methanoculleus marisnigri* JR1 of the order of *Methanomicrobiales* became available [51]. To gain an insight into the relatedness of dominant methanogens within the analysed fermentation sample to this reference species, several genes were analysed in more detail.

**Figure 5:**
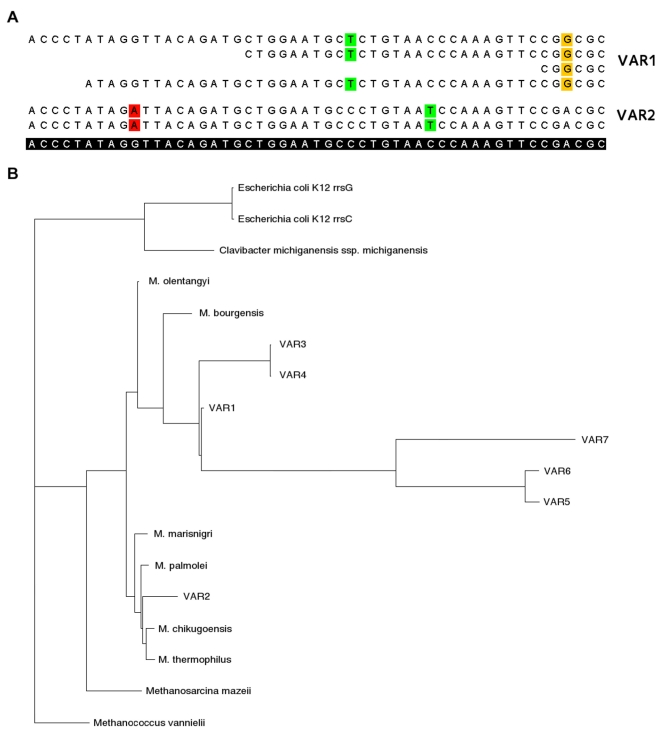
Identification of *Methanoculleus* variants. Partial view (A) of pyrosequencing reads aligned to the 16S rRNA sequence of *Methanoculleus bourgensis* (Genbank accession AY196674). Colored bases indicate differences between reads and the reference (shown in the bottom line). In the depicted part, two of the seven different variants are visible. To characterize the variants, a phylogenetic tree (B) was constructed together with various reference sequences. Most variants show close relationship to *M. bourgensis*; only variant VAR2 was placed in another branch formed by *M. marisnigri*, *M. palmolei*, *M. chikugoensis* and *M. thermophilus*. Several 16S rRNA sequences from the genus *Methanoculleus* were used: *M. olentangyi* (AF095270), *M. bourgensis* (AY196674), *M. palmaeoli* (Y16382), *M. thermophilus* (AB065297), *M. chikugoensis* MG62 (AB038795) and *M. marisnigri* JR1 (CP000562 (Memar_R0043)). Additional sequences in increasing taxonomic distance were included as outgroups: *Methanosarcina mazeii* (MMU20151), *Methanococcus vannielii* SB (CP000742 (Mevan_R0025)), *Clavibacter michiganensis ssp. michiganensis* NCPPB 382 (AM711867 (CMM_RNA_0001)) and two sequences from *Escherichia coli* K12 DH10B (NC_010473 (ECDH10B_3945 and ECDH10B_2759)).

From both the (unfiltered) GS FLX and Titanium datasets, a total amount of 5,266 reads containing 16S rRNA sequence fragments was identified. Out of these, 44 of the GS FLX and 88 of the Titanium reads were taxonomically assigned to the genus *Methanoculleus* by the RDP classifier, but could not be assembled into a single consensus sequence due to differences in sequence composition. Binning of the individual reads based on their SNP content (Figure 5A) and subsequent assembly resulted in seven consensus sequences, each of which comprised at least the partial sequence of a specific 16S rRNA gene. Subsequently, the consensus sequences were characterized in terms of their phylogeny together with several reference sequences obtained from GenBank (Figure 5B). All sequences except one were placed in close phylogenetic distance to *Methanoculleus bourgensis*; the one remaining sequence was placed in a remote branch formed by *Methanoculleus marisnigri*, *Methanoculleus palmolei*, *Methanoculleus chikugoensis* and *Methanoculleus thermophilus* (Figure 5B). These findings are consistent with the results of a previous study [35], where a phylogenetic characterization of the same biogas plant based on 16S rRNA clone library sequences was performed.

For verification, the same analysis was repeated for the 5S rRNA and the *mtrB* gene. While the *mcr* operon is partially duplicated in the genome of *Methanoculleus marisnigri* JR1, only one copy of the *mtrB* gene exists. Four different variants of the *mtrB* gene could be assembled, confirming the presence of several *Methanoculleus* species/strains (see File S1). Assembly of the 5S rRNA gene confirmed three variants (data not shown); downstream of the 5S rRNA gene, all three variants differ from *Methanoculleus marisnigri* JR1, indicating a different genomic context for the *rrn* cluster.

### Mapping of metagenome reads to the *Methanoculleus marisnigri* JR1 reference genome

In an attempt to reconstruct the genome sequence of one of the dominant methanogenic species, both datasets were mapped onto the published genome of *Methanoculleus marisnigri* JR1, the only *Methanoculleus* strain for which a completely sequenced genome currently exists. Using only the sequence data from the GS FLX dataset, 39.8% of the reference genome was covered; mapping of the Titanium dataset resulted in 41.7% coverage. A joint mapping of both datasets produced a genomic coverage of 45.4%.

**Figure 6:**
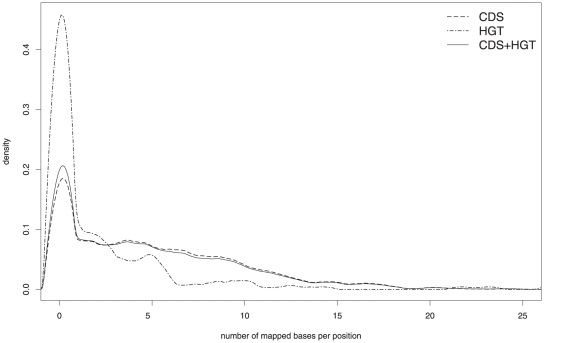
Comparison of kernel density estimates for metagenome reads mapped to coding regions of the *Methanoculleus marisnigri* JR1 genome. The figure shows the mapped bases per position in the genome separated into different groups: genes that have potentially been acquired by horizontal gene transfer (HGT, depicted as dot-dashed line) and all other genes without a prediction for horizontal gene transfer (CDS, shown as dashed line). For comparison, the density function for all genes combined is shown as well (solid line). Poor coverage of HGT genes hints at genomic features probably missing in the *Methanoculleus* species present in the studied biogas fermenter, further supporting their difference to *Methanoculleus marisnigri* JR1.

Upon further analysis, a correlation between poorly covered regions and areas with relatively low GC content was discovered (data not shown). Since variations in GC content often hint at horizontal gene transfer, the number of bases that could be mapped to the coding sequence of each gene in the genome of *Methanoculleus marisnigri* JR1 was determined and normalized with respect to the gene length. The results were divided into two different groups depending on the prediction found in the HGT-DB: one containing all genes that potentially have been acquired by horizontal gene transfer, and another one for all remaining genes. For both groups, the density function of all results was plotted (Figure 6). A comparison of the results for genes predicted as acquired by horizontal gene transfer in the HGT-DB and the remaining genes suggests that some mobile DNA segments are missing in one or several of the *Methanoculleus* species present in the studied biogas fermenter. The coverage of the *Methanoculleus marisnigri* JR1 genome as determined from the available metagenome sequence data does not suffice to determine the absence or presence of individual genes in the *Methanoculleus* species residing in the biogas fermenter. This is particularly relevant because several different *Methanoculleus* species/strains were identified in the studied biogas fermenter. Even with the currently available sequence data, the reconstruction of a complete genome of one of the dominant species still remains unfeasible due to the complexity of the microbial community and the close relationship of some of the dominant species.

### Hydrogenases of Methanomicrobiales

This study revealed that species of the genus *Methanoculleus* dominate among methanogenic *Archaea*. High abundance of *Methanoculleus* members has also been shown for other communities involved in fermentation of maize silage and related substrates [58], [68], [72], [73] suggesting that in these habitats methane is mainly produced via the hydrogenotrophic pathway by reduction of carbon dioxide. Hydrogenotrophic methanogenesis usually is accompanied by syntrophic acetate oxidation which necessitates that released hydrogen from acetate oxidation is efficiently consumed by cooperating hydrogenotrophic methanogens. It is assumed that *Methanoculleus* species have a high hydrogen affinity [73]. BLAST analyses revealed that several metagenome reads from the biogas community correspond to *M. marisnigri* JR1 hydrogenase genes (see Table 2). Among the identified genes are those possibly encoding the membrane-bound hydrogenases Eha (Memar_1172 - Memar_1185) and Ech (Memar_0359 - Memar_0364), which were predicted to participate in methanogenesis [51]. It is likely that Eha and Ech represent high-affinity hydrogenases contributing to efficient hydrogen oxidation in the course of methanogenesis. At least some *Methanoculleus* members within the community of the analysed fermentation sample possess membrane-bound hydrogenases related to Eha and Ech of *Methanoculleus marisnigri* JR1.

**Table 2:** Metagenome reads comprising hydrogenase gene fragments.

Titanium^a^	GS FLX^a^	locus	description
462	134	Memar_0359^b^	4Fe-4S ferredoxin iron-sulfur binding domain-containing protein
66	44	Memar_0360^b^	NADH-ubiquinone oxidoreductase, chain 49kDa
14	16	Memar_0361^b^	ech hydrogenase, subunit EchD, putative
40	17	Memar_0362^b^	NADH ubiquinone oxidoreductase, 20 kDa subunit
221	111	Memar_0417	(NiFe) hydrogenase maturation protein HypF
46	32	Memar_0470	hydrogenase accessory protein HypB
111	46	Memar_0622	methyl-viologen-reducing hydrogenase, delta subunit
80	59	Memar_1007^c^	nickel-dependent hydrogenase, large subunit
55	28	Memar_1008^c^	NADH ubiquinone oxidoreductase, 20 kDa subunit
21	21	Memar_1014	hydrogenase maturation protease
146	51	Memar_1022	hydrogenase expression/formation protein HypE
11	10	Memar_1023	hydrogenase expression/synthesis, HypA
16	7	Memar_1024	hydrogenase assembly chaperone hypC/hupF
87	49	Memar_1044	coenzyme F420 hydrogenase/dehydrogenase beta subunit
72	45	Memar_1140	hydrogenase expression/formation protein HypD
21	24	Memar_1172^d^	hypothetical protein
12	8	Memar_1173^d^	hypothetical protein
14	10	Memar_1174^d^	hypothetical protein
18	5	Memar_1175^d^	hypothetical protein
37	18	Memar_1176^d^	hypothetical protein
18	15	Memar_1177^d^	uncharacterized membrane protein
3	6	Memar_1179^d^	hypothetical protein
3	5	Memar_1181^d^	hypothetical protein
28	13	Memar_1182^d^	hypothetical protein
24	14	Memar_1183^d^	NADH ubiquinone oxidoreductase, 20 kDa subunit
281	68	Memar_1185^d^	4Fe-4S ferredoxin iron-sulfur binding domain-containing protein
49	38	Memar_1378	coenzyme F420 hydrogenase/dehydrogenase beta subunit
17	6	Memar_1380	coenzyme F420 hydrogenase/dehydrogenase beta subunit
79	43	Memar_1623	coenzyme F420 hydrogenase/dehydrogenase beta subunit
93	45	Memar_2174	nickel-dependent hydrogenase, large subunit
31	21	Memar_2175	hydrogenase maturation protease
544	178	Memar_2176	coenzyme F420 hydrogenase
54	29	Memar_2177	coenzyme F420-reducing hydrogenase subunit beta

a number of reads assigned to a specific *M. marisnigri* JR1 locus (Memar);

b Ech operon encoding a membrane-bound hydrogenase [51];

c F_420_ non-reducing hydrogenase; d Eha operon encoding a membrane-bound hydrogenase [51].

### Coverage of the microbial community

To analyse whether the diversity of the biogas-producing microbial community is sufficiently covered by the sequence data, species richness, diversity and rarefaction calculations were conducted.

The Shannon index [74] was used to estimate the biological diversity of the underlying microbial community in the analyzed fermenter. It is widely applied in ecological studies as a measurement of biodiversity, accounting for both the number of different taxa as well as their relative abundances. For this purpose, 16S rRNA fragments were detected in the normalized pyrosequencing datasets and classified with the RDP classifier. Subsequently the Shannon index was computed considering only 16S rRNA sequences that could be classified on rank genus with at least 80% confidence.

This approach resulted in a Shannon index value of 1.90 for the GS FLX and 2.51 for the Titanium dataset, showing that sequence data obtained from the GS FLX platform clearly underestimates the biodiversity of the underlying community.

Since both datasets differ in size, the Shannon index was also computed for random subsets of 407,558 reads (i.e. the size of the normalized GS FLX dataset) extracted from the Titanium data; 5,000 iterations were calculated and the average of all results taken. The resulting index value of 2.58 shows that even for equally sized datasets, the Titanium dataset provides an higher estimate of biodiversity.

**Figure 7:**
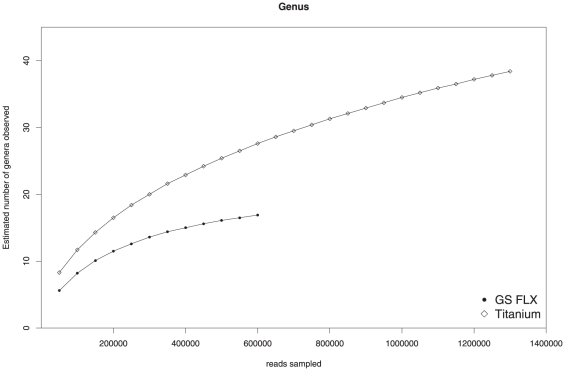
Rarefaction analysis of observed genera. The rarefaction curves represent the estimated number of genera that would be observed in biogas fermenter metagenomes of different sizes. The values were determined based on 16S rRNA fragments classified at rank genus identified in the entire Titanium and GS FLX datasets, respectively.

Rarefaction analyses of both unfiltered datasets revealed that the biogas-producing community is covered much deeper by the Titanium than by the GS FLX sequences (Figure 7). 16S rRNA fragments identified in the Titanium sequences were assigned to 38 different genera, but only 16 genera were observed for the GS FLX dataset. Three genera were specific for the GS FLX dataset, 13 were shared, and 25 were specific for the Titanium data. A full list of all identified genera can be found in the File S1. The rarefaction curve computed for the Titanium dataset was also far from reaching the plateau phase, indicating that considerably higher sequencing effort would be required to cover all phylogenetic groups of the underlying microbial community. Rarefaction analysis conducted at rank family is in accordance with this conclusion (see File S1).

**Figure 8:**
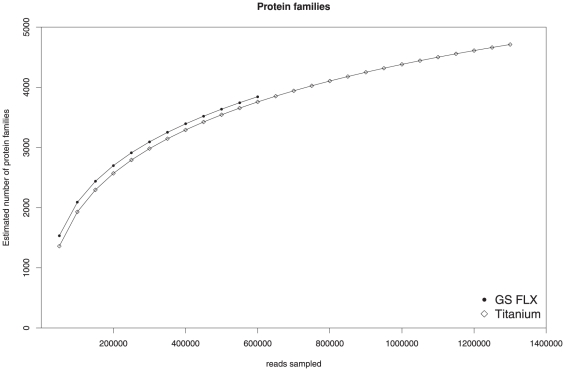
Rarefaction analysis of Pfam families. The estimated number of Pfam protein families that would be identified in biogas fermenter metagenomes of different sizes is shown. The values were computed based on the number of protein families identified in the entire Titanium and FLX metagenomes, respectively.

The gene content of the studied metagenome samples was characterized by assigning reads to Pfam protein families. As expected, the collective gene content of the studied microbial community was covered much deeper by the Titanium than by the GS FLX sequence reads. In total, 4,759 different protein families were identified in the Titanium dataset, while 3,844 could be detected in the GS FLX reads. However, the rarefaction curves computed for the number of protein families also did not reach the plateau phase, suggesting that additional sampling would be required to capture the entire gene content of the underlying community (Figure 8). Protein families identified in the Titanium reads were annotated with 1,623 different GO terms, whereas 1,338 GO terms were observed for the GS FLX dataset. Compared to the GS FLX data, the Titanium dataset provides a far more detailed overview of the gene content and metabolic potential of the studied microbial community.

## Discussion

In a recent study, the metagenome of a biogas-producing microbial community was sequenced employing the GS FLX pyrosequencing platform. Since analysis results showed insufficient coverage of the community, the study was complemented by additional sequencing of the same DNA preparation on the GS FLX Titanium platform.

During the analysis, a previously unreported GC bias in pyrosequencing data was identified, which affected sequences from both sequencing runs, thus indicating the importance of thorough data screening and filtering to avoid a contortion of results. However, sequence data generated using the GS FLX Titanium chemistry was only marginally affected by this issue and only a very small fraction of reads had to be excluded from further analysis. These differences result from improvements in the Titanium chemistry, leading to less bias compared to the previous GS FLX technology. Meanwhile, a new emPCR kit containing a specific additive has been launched by Roche Applied Science. Initial studies based on microbial genome sequencing revealed an almost bias free sequencing of even very high GC content regions (data not shown).

The composition of the microbial community was deduced from both the taxonomic classification of 16S rRNA fragments as well as the assignment of Environmental Gene Tags (EGTs) on different taxonomic ranks Obtained results essentially confirmed taxonomic profiles of the previous study. However, less abundant taxa could be identified by analyzing the GS FLX Titanium dataset thus justifying the additional sequencing effort. The fact that only a very small fraction of metagenomic reads actually contains fragments of the 16S rRNA gene emphasizes the advantages of using software such as the CARMA pipeline, which accurately classifies gene fragments detected in metagenome sequence data. A rarefaction analysis was performed to estimate the coverage of the microbial community in both sequencing datasets; as expected, sequencing data from the GS FLX Titanium platform provides a far more complete view of the underlying community, while the GS FLX sequencing run was not carried out to saturation.

During the functional characterization of the community, members of the phylum *Firmicutes* could be confirmed to represent the dominant organisms involved in the breakdown of polysaccharides together with *Bacteroidetes*. Beyond that, the novel analyses showed that *Proteobacteria* also play an important role in polysaccharide degradation. *Clostridia* were found to dominate within the functional context ‘acetogenesis’, as deduced by mapping of bacterial taxa to metagenome hits representing the Wood-Ljungdahl pathway which is also known as the reductive acetyl-CoA pathway. *Methanomicrobiales* are the most abundant order involved in methanogenesis using CO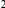 as a carbon source, while acetate only seems to play a minor role, as indicated by a low fraction of *Methanosarcinales*.

Based on the identification of 16S rRNA fragments from the *Methanoculleus* genus and subsequent assembly, the presence of several *Methanoculleus* species closely related to *Methanoculleus bourgensis* in the studied biogas fermenter could be demonstrated. A rough characterization of the genomic content of these *Methanoculleus* species was conducted by mapping the metagenome sequence reads to the published genome of *Methanoculleus marisnigri* JR1. Comparison of the genomic content of dominant *Methanoculleus* species within the analysed sample and the reference species *M. marisnigri* JR1 revealed that there are several differences mainly concerning genes that might have been acquired by horizontal gene transfer. Metagenome reads assigned to the genus *Methanoculleus* represent *inter alia* methanogenesis and membrane-bound hydrogenase genes predicted to be of importance for the pathway leading to the formation of methane within biogas. The close relationship of the *Methanoculleus* species in the studied biogas fermenter makes reconstruction of the genomic sequence of one of the dominant *Methanoculleus* species from the metagenomic sequence reads rather unlikely, since a reliable distinction between the most abundant strains can not be assured. In comparison, the GS FLX Titanium data offers a far more complete view of the analysed fermenter, even though analysis results give evidence that the available sequence data still does not fully cover the microbial community.

## Supporting Information

File S1 Supporting material.
(0.26 MB PDF)
