# Supplementary material for: Comparative and Joint Analysis of Two Metagenomic Datasets from a Biogas Fermenter Obtained by 454-Pyrosequencing
Source: PLoS One. 2011 Jan 26;6(1):e14519. doi: 10.1371/journal.pone.0014519 (PMC3027613; doi:10.1371/journal.pone.0014519)
Supplement: File S1 — Supporting material. (0.26 MB PDF) [file pone.0014519.s001.pdf]

CeBiTec
Center for Biotechnology

Comparative and joint analysis of two metagenomic datasets from a biogas fermenter obtained by 454-pyrosequencing

– Supplemental material –

1 Read length distribution of pyrosequencing runs

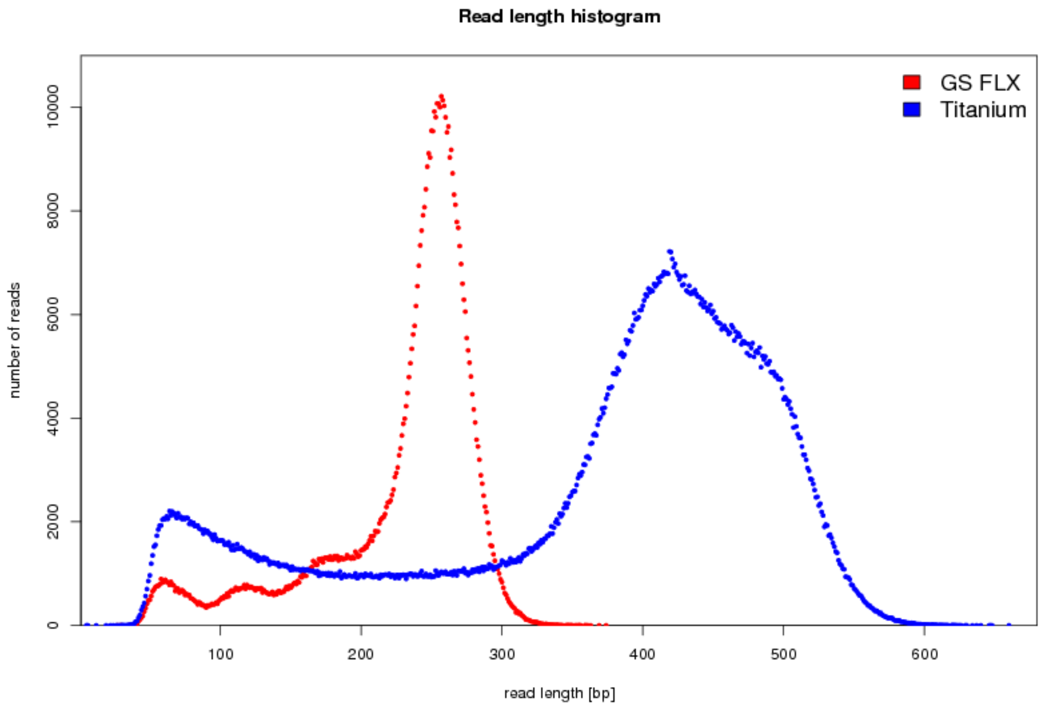

Figure 1.1: **Read length histogram.** 616,072 reads with an average length of 230.0 bp were obtained from the GS FLX run (red points), while the GS FLX Titanium dataset (blue points) consists of 1,347,644 reads with an average length of 367.7 bp.

2 GC bias in pyrosequencing data

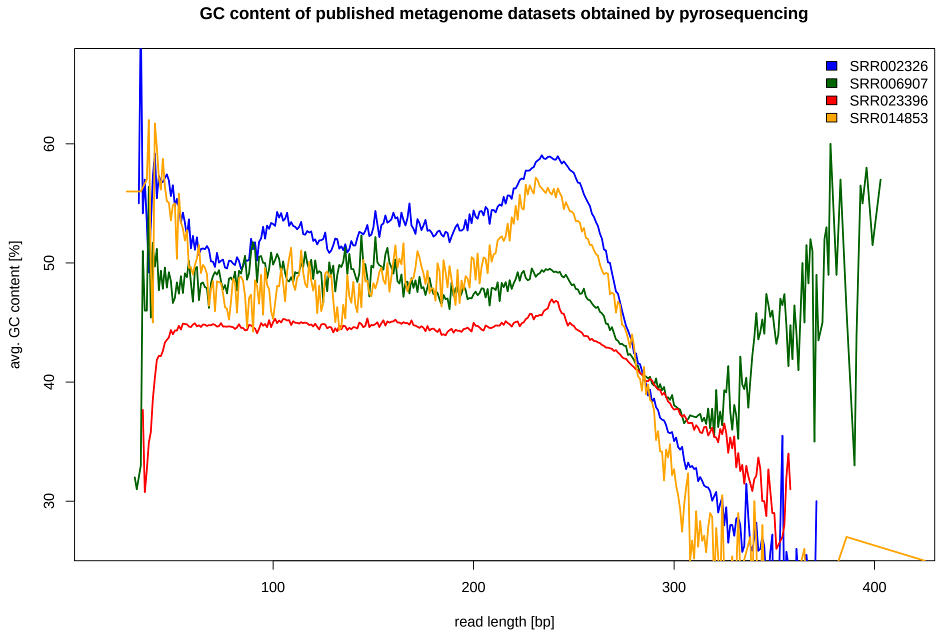

Figure 2.1: **GC bias in published metagenome sequences downloaded from NCBI's Short Read Archive (SRA).** The analysis was conducted for data from a glacier ice metagenome (SRR002326), a freshwater metagenome (SRR006907), the Brazos-Trinity basin sediment metagenome (SRR023396) and the Windshield splatter metagenome (SRR014853). All analysed metagenome datasets showed a GC bias similar to the one detected for both biogas fermenter metagenome datasets.

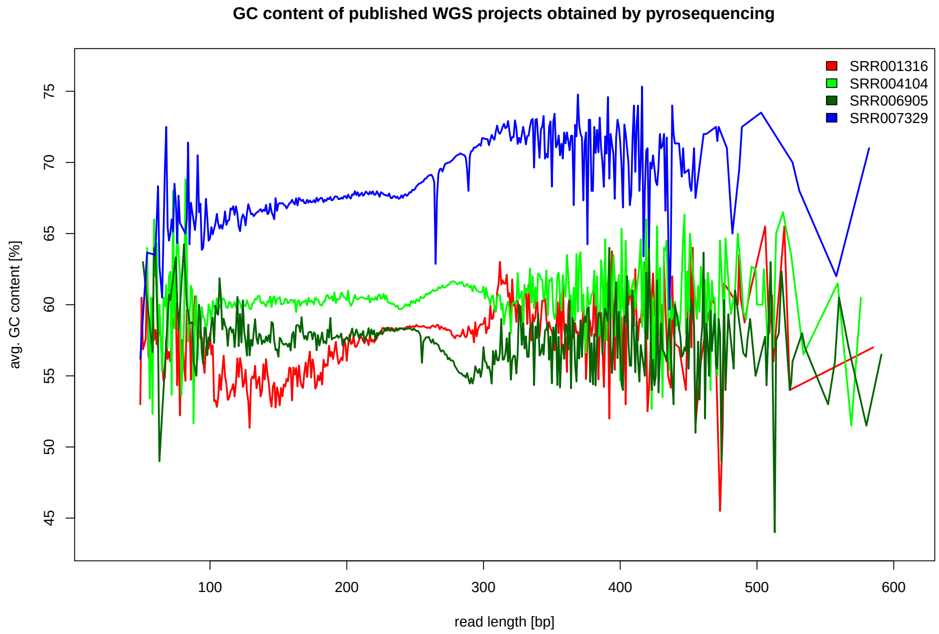

Figure 2.2: **Verification of GC bias analysis for published genome project reads downloaded from NCBI's Short Read Archive (SRA).** The analysis was repeated for sequence reads from WGS sequencing projects, where the GC bias cannot be observed due to limited distribution of GC content across all reads. Data shown represents sequences from *Bifidobacterium dentium* ATCC 27678 (SRR001316), *Geobacter sp.* M21 (SRR004104), *Rhodobacteraceae bacterium* KLH11 PRK11 (SRR006905) and *Streptomyces sp.* H668 PS668 (SRR007329). Several other public datasets were analyzed (data not shown), which yielded similar results.

3 Functional analyses

Table 3.1: COG entries used for the classification of reads on taxonomic and physiological level.

**POLYSACCHARIDE DEGRADATION**	
CELLULOSE	
COG1363	Cellulase M and related proteins
COG2730	Endoglucanase
COG3405	Endoglucanase Y
COG1472	β-glucosidase-related glycosidases
COG4124	β-Mannanase
COG1447	Phosphotransferase system cellobiose-specific component IIA
COG3459	Cellobiose phosphorylase
COG5297	Cellobiohydrolase A (1,4-β-cellobiosidase A)
COG5309	Exo-beta-1,3-glucanase
COG0366	Glycosidases
COG1874	β-galactosidase
COG3250	β-galactosidase/β-glucuronidase
HEMICELLULOSE (XYLAN)	
COG2115	Xylose isomerase
COG4213	ABC-type xylose transport system, periplasmic component
COG3693	β-1,4-xylanase
COG3507	β-xylosidase
COG4677	Pectin methylesterase
NOG13929	Endo 1,4-β Xylanase
NOG06229	Xylosidase
COG5434	Endopolygalacturonase
COG3866	Pectate lyase
COG3867	Arabinogalactan endo-1,4-β-galactosidase
LIGNIN	
COG2132	Putative multicopper oxidases
COG1496	Uncharacterized conserved protein (pfam02578)

ACETOGENESIS/METHANOGENESIS

METHANOGENESIS/ACETATE

(Firmicutes)

COG0282	Acetate kinase
COG4002	Predicted phosphotransacetylase
COG0280	Phosphotransacetylase

(Methanosarcinales)

COG1152	CO dehydrogenase/acetyl-CoA synthase alpha subunit
COG1456	CO dehydrogenase/acetyl-CoA synthase gamma subunit (corrinoid Fe-S protein)
COG1880	CO dehydrogenase/acetyl-CoA synthase epsilon subunit
COG1614	CO dehydrogenase/acetyl-CoA synthase beta subunit
COG2069	CO dehydrogenase/acetyl-CoA synthase delta subunit (corrinoid Fe-S protein)

METHANOGENESIS/CO2

COG1229	Formylmethanofuran dehydrogenase subunit A (Memar_0371, Memar_0619, Memar_1188)
COG1029	Formylmethanofuran dehydrogenase subunit B
COG2218	Formylmethanofuran dehydrogenase subunit C
COG1153	Formylmethanofuran dehydrogenase subunit D (Memar_1186, Memar_0373, Memar_0621)
COG2191	Formylmethanofuran dehydrogenase subunit E (Memar_1046, Memar_2371)
COG2037	Formylmethanofuran tetrahydromethanopterin formyltransferase (Memar_0628)
COG4074	H2-forming N5,N10-methylenetetrahydromethanopterin dehydrogenase
COG4063	Tetrahydromethanopterin S-methyltransferase, subunit A (Memar_0606, Memar_0608)
COG4062	Tetrahydromethanopterin S-methyltransferase, subunit B (Memar_0609)
COG4061	Tetrahydromethanopterin S-methyltransferase, subunit C (Memar_0610)
COG4060	Tetrahydromethanopterin S-methyltransferase, subunit D (Memar_0611)
COG4059	Tetrahydromethanopterin S-methyltransferase, subunit E (Memar_0607)
COG4118	Tetrahydromethanopterin S-methyltransferase, subunit F
COG4064	Tetrahydromethanopterin S-methyltransferase, subunit G
COG3252	Methenyltetrahydromethanopterin cyclohydrolase

4 Biodiversity and Rarefaction

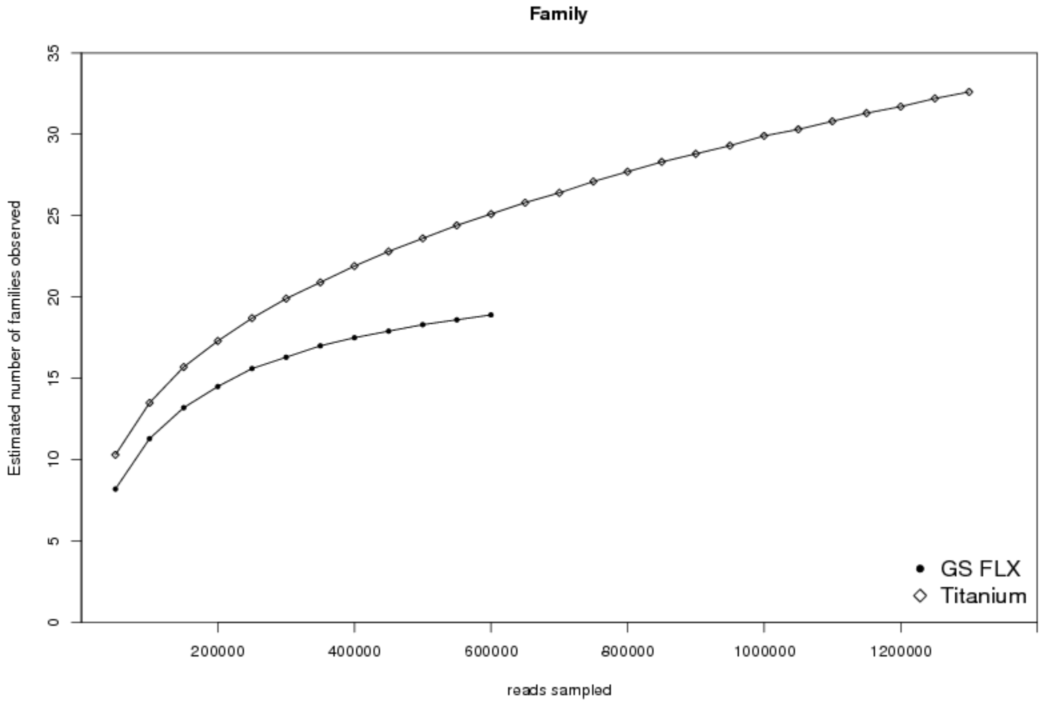

Figure 4.1: **Rarefaction analysis of observed families.** The estimated number of families that was observed for biogas fermenter metagenomes of different sizes is presented. The values were estimated based on the number of families identified for the entire Titanium and FLX metagenomes, respectively. Fragments of 16S rDNAs detected in the metagenomes were assigned on family level using the RDP classifier.

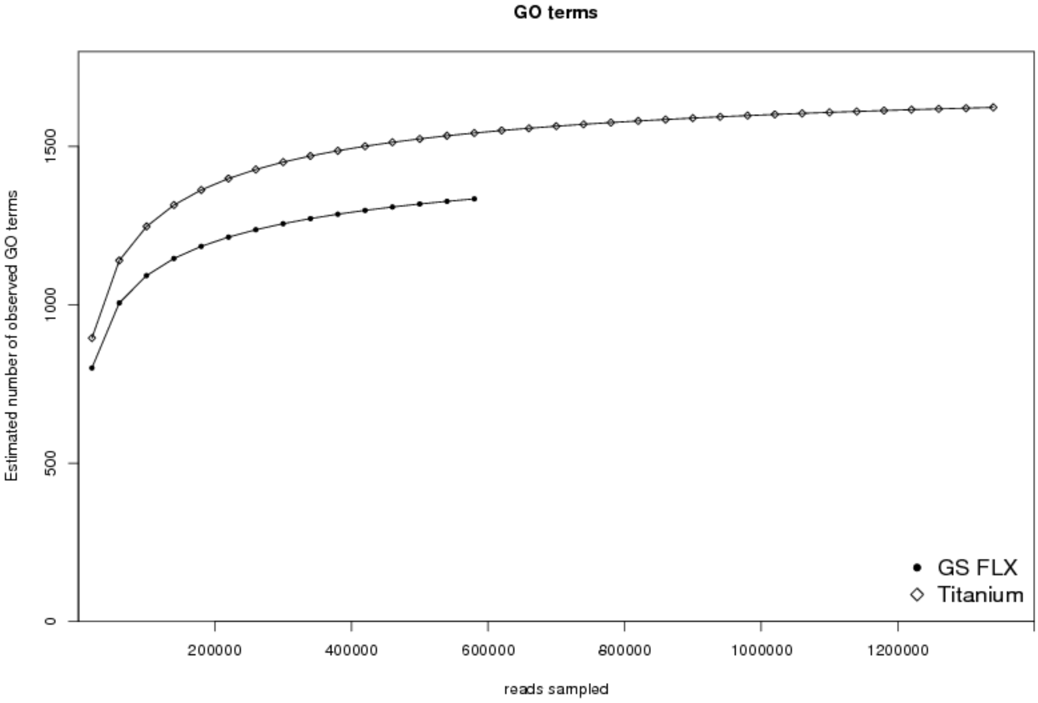

Figure 4.2: **Rarefaction analysis of GO terms.** Depicted is the estimated number of GO terms that would be identified in biogas fermenter metagenomes of different sizes. The values were estimated based on the number of GO terms identified in the entire Titanium and FLX metagenomes, respectively.

Phylum	Genus	Titanium	GS FLX
Euryarchaeota	Methanoculleus	84	44
Firmicutes	Clostridium	62	11
	Sedimentibacter	58	7
	Streptococcus	14	0
	Syntrophomonas	9	8
	Acetivibrio	8	0
	Anaerovorax	7	2
	Ruminococcus	5	1
	Thermacetogenium	3	1
	Garciella	3	0
	Tissierella	2	0
	Gracilibacter	2	0
	Gelria	2	0
	Turicibacter	1	0
	Thermosediminibacter	1	0
	Thermoanaerobacter	1	0
	Syntrophothermus	1	0
	Sporobacter	1	0
	Pelotomaculum	1	2
	Lactobacillus	1	0
	Halocella	1	0
	Enterococcus	1	0
	Desulfonispora	1	0
	Dehalobacter	1	0
	Abiotrophia	1	0
	Holdemania	0	1
Tenericutes	Acholeplasma	44	5
	Phytoplasma	2	0
Bacteroidetes	Proteiniphilum	13	3
	Petrimonas	9	4
	Dysgonomonas	3	0
	Alkaliflexus	3	0
	Niastella	1	0
Synergistetes	Anaerobaculum	5	2
Spirochaetes	Treponema	3	2
Actinobacteria	Bifidobacterium	2	0
	Tetrasphaera	0	1
Planctomycetes	Planctomyces	1	0
Proteobacteria	Bordetella	1	0
	Arcobacter	1	0
Thermotogae	Petrotoga	0	4

Table 4.1: **Total number of genera identified in the Biogas fermenter.** Listed are all genera identified by classification of 16S rDNA fragments together with their absolute number that were detected in one of the pyrosequencing datasets. Genera are grouped by their corresponding phylum and sorted based on the number of assigned 16S rDNA fragments detected for the GS FLX Titanium data.

5 Identification of several different Methanoculleus species

mtrB

The *mcr* genes encoding enzymes of the methanogenesis pathway (http://www.genome.jp/dbget-bin/get_pathway?org_name=mem&mapno=00790) are (partially) duplicated occurring at nucleotide positions 354913-359266 and 584671-586578 in the genome of *Methanoculleus marisnigri* JR1. A further component of the *mcrA* complex encoding an ABC transporter-like ATPase is located at position 2055559-2057122 (Memar_2065; *mcrA2*).

gene	1. cluster	2. cluster	identity	domain
mcrB	Memar_0375	Memar_0617		pfam02783 and pfam02241,
	YP_001046290	YP_001046532		MCR_beta_N, Methyl-coenzyme M
	434 aa	434 aa	352/433 (81%)	reductase beta subunit, N- and
	1305 bp	1305 bp	1072/1299 (82%)	C-terminal domain; COG4054
mcrD	Memar_0376	Memar_0616		pfam02505, MCR_D,
	YP_001046291	YP_001046531		Methyl-coenzyme M
	159 aa	158 aa	86/155 (55%)	reductase operon protein D;
	480 bp	477 bp	205/305 (67%)	COG4055
mcrC	-	Memar_0615		pfam04609, MCR_C,
		YP_001046530		Methyl-coenzyme M
		207 aa	-	reductase operon protein C;
		626 bp	-	COG4056
mcrG	Memar_0377	Memar_0614		pfam02240, MCR_gamma,
	YP_001046292	YP_001046529		Methyl-coenzyme M
	254 aa	254 aa	224/253 (88%)	reductase gamma subunit;
	765 bp	765 bp	651/760 (85%)	COG4057
mcrA	Memar_0378	Memar_0613		pfam02745 and pfam02249,
	YP_001046293	YP_001046528		MCR_alpha_N, Methyl-
	568 aa	568 aa	503/568 (88%)	coenzyme M reductase,
	1707 bp	1707 bp	1518/1707 (88%)	alpha subunit, N- and C-
				terminal domain; COG4058

Table 5.1: **Gene duplication in the *mcr* operon of *Methanoculleus marisnigri* JR1.**

The gene Memar_0609 (*mtrB*; 285 nt, 94 aa) encodes a subunit of the tetrahydromethanopterin S-methyltransferase (pfam05440, MtrB, Tetrahydromethanopterin S-methyltransferase subunit B; COG4062) involved in the biosynthesis of N5-methyltetrahydromethanopterin coenzyme M. Only one copy of the gene is present in the genome. Figure 5.1 shows reads from both datasets aligned to the reference sequence of Memar_0609, with SNPs highlighted in different colors. Four different variants of the *mtrB* gene can be distinguished.

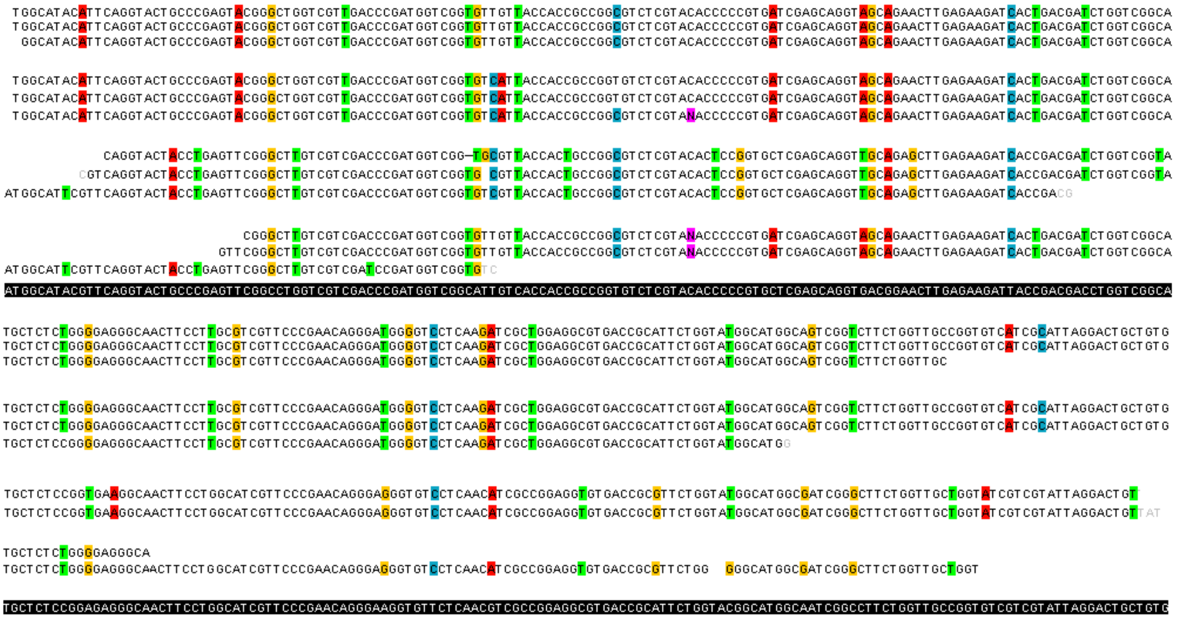

Figure 5.1: **Detail view of metagenome reads aligned to the nucleotide sequence of Memar_0609 (*mtrB*).** The aligned reads have been grouped based on SNPs, showing four different variants.
